# A phase 1b randomized clinical trial of CT1812 to measure Aβ oligomer displacement in Alzheimer’s disease using an indwelling CSF catheter

**DOI:** 10.1186/s40035-023-00358-w

**Published:** 2023-05-12

**Authors:** Kelsie M. LaBarbera, Yvette I. Sheline, Nicholas J. Izzo, Carla M. Yuede, Lora Waybright, Raymond Yurko, Hannah M. Edwards, Woodrow D. Gardiner, Kaj Blennow, Henrik Zetterberg, Anne Börjesson-Hanson, Roger Morgan, Charles S. Davis, Robert J. Guttendorf, Lon S. Schneider, Steven DeKosky, Harry LeVine, Michael Grundman, Anthony O. Caggiano, John R. Cirrito, Susan M. Catalano, Mary E. Hamby

**Affiliations:** 1 Cognition Therapeutics Inc., Pittsburgh, PA USA; 2 University of Pennsylvania, Philadelphia, USA; 3 Washington University, St. Louis, USA; 4 University of Gothenburg, Mölndal, Sweden; 5 Department of Psychiatry and Neurochemistry, Institute of Neuroscience and Physiology, The Sahlgrenska Academy at the University of Gothenburg, Mölndal, Sweden; 6 Clinical Neurochemistry Laboratory, Sahlgrenska University Hospital, Mölndal, Sweden; 7 Department of Neurodegenerative Disease, UCL Institute of Neurology, Queen Square, London, UK; 8 UK Dementia Research Institute at UCL, London, UK; 9 Hong Kong Center for Neurodegenerative Diseases, Clear Water Bay, Hong Kong China; 10 Wisconsin Alzheimer’s Disease Research Center, University of Wisconsin School of Medicine and Public Health, University of Wisconsin-Madison, Madison, WI USA; 11 Karolinska University Hospital, Stockholm, Sweden; 12 MedSurgPI, LLC, Raleigh, NC USA; 13 CSD Biostatistics, Inc., Oro Valley, AZ USA; 14 Aclairo Pharmaceutical Development Group, Inc, Vienna, VA USA; 15 Keck School of Medicine of USC, Los Angeles, CA USA; 16 McKnight Brain Institute, University of Florida, Gainesville, FL USA; 17 Sanders-Brown Center on Aging, University of Kentucky, Lexington, KY USA; 18 Global R&D Partners, LLC, San Diego, CA USA; 19 Department of Neurosciences, University of California, San Diego, USA

*Trial Registration*: May 11th, 2018 ClinicalTrials.gov Identifier: NCT03522129 https://clinicaltrials.gov/ct2/show/NCT03522129.

Investigational therapies for Alzheimer's disease (AD) target a wide range of mechanisms, yet promising disease-modifying therapies remain a huge unmet need. Much evidence indicates that the oligomeric form of amyloid-beta (Aβ) is a toxic species contributing to AD through synaptic damage and neuronal toxicity [1]. In support of this, Aβ oligomer reduction in an AD mouse model leads to memory preservation [2, 3], and clinical benefit was observed in trials of lecanemab, which targets Aβ oligomers and protofibrils [4], in AD patients, encouraging the continued development of Aβ oligomer-targeting therapies.

CT1812 is a novel, small-molecule, brain-penetrant sigma-2 receptor (S2R) modulator that selectively prevents and displaces Aβ oligomers from binding to neuronal synapses, thereby mitigating downstream toxicity. This is thought to occur through allosteric modulation of the oligomer receptor, cellular prion protein (PrP^c^), by direct interaction between PrP^c^ and S2R at synapses [3, 5]. CT1812 rescues synaptic loss and neuronal function in primary neuronal cultures, and restores cognition in a mouse model of AD to levels of healthy controls [3].

Demonstration of target engagement, successful binding of a compound to its intended receptor, is important for increasing probability of success in drug development. Given the mechanism of action of CT1812, we postulated that Aβ oligomers could be a proximal indicator of S2R target engagement. Preclinical studies supported this, with CT1812 impacting Aβ oligomers in AD transgenic mice [3]. Aβ oligomer levels in hippocampal interstitial fluid and lateral ventricle cerebrospinal fluid (CSF) were measured in vivo using a microimmunoelectrode (MIE) coated with an oligomer-selective antibody following a single administration of CT1812. A significant, dose-dependent rise in Aβ oligomers was detected after administration, consistent with Aβ oligomer displacement from neurons and clearance into CSF. In contrast, Aβ monomer levels were not affected [3].

To determine whether this approach could demonstrate target engagement in patients with AD, a phase 1b, randomized, double-blind, placebo-controlled trial (NCT03522129) was designed. Eligibility criteria for the study included a diagnosis of mild-moderate AD (Mini-Mental State Examination score 18–26) and either an AD-positive amyloid positron emission tomography scan or a positive CSF result for AD biomarkers (Additional file 1) within 12 months before or at screening. Participants were randomized 2:1 to receive either a single oral dose of CT1812 (560 mg) or identically appearing placebo capsules. An indwelling catheter was placed in the lumbar subarachnoid space and 4–6 ml CSF samples were collected hourly for 28 h (five samples pre-dose and 24 samples following drug or placebo administration). Plasma was taken at the same intervals for pharmacokinetic analysis.

Although intended enrollment was 18 patients, recruitment was challenged by the 28-h spinal catheter procedure and the single-dose nature of the study without optional open-label extension. Fifteen participants were screened and three were randomized and completed the trial before it was ceased. Baseline demographics are shown in Fig. 1a. There were no deaths and no subjects were withdrawn from the study due to treatment-emergent adverse events.

**Fig. 1:**
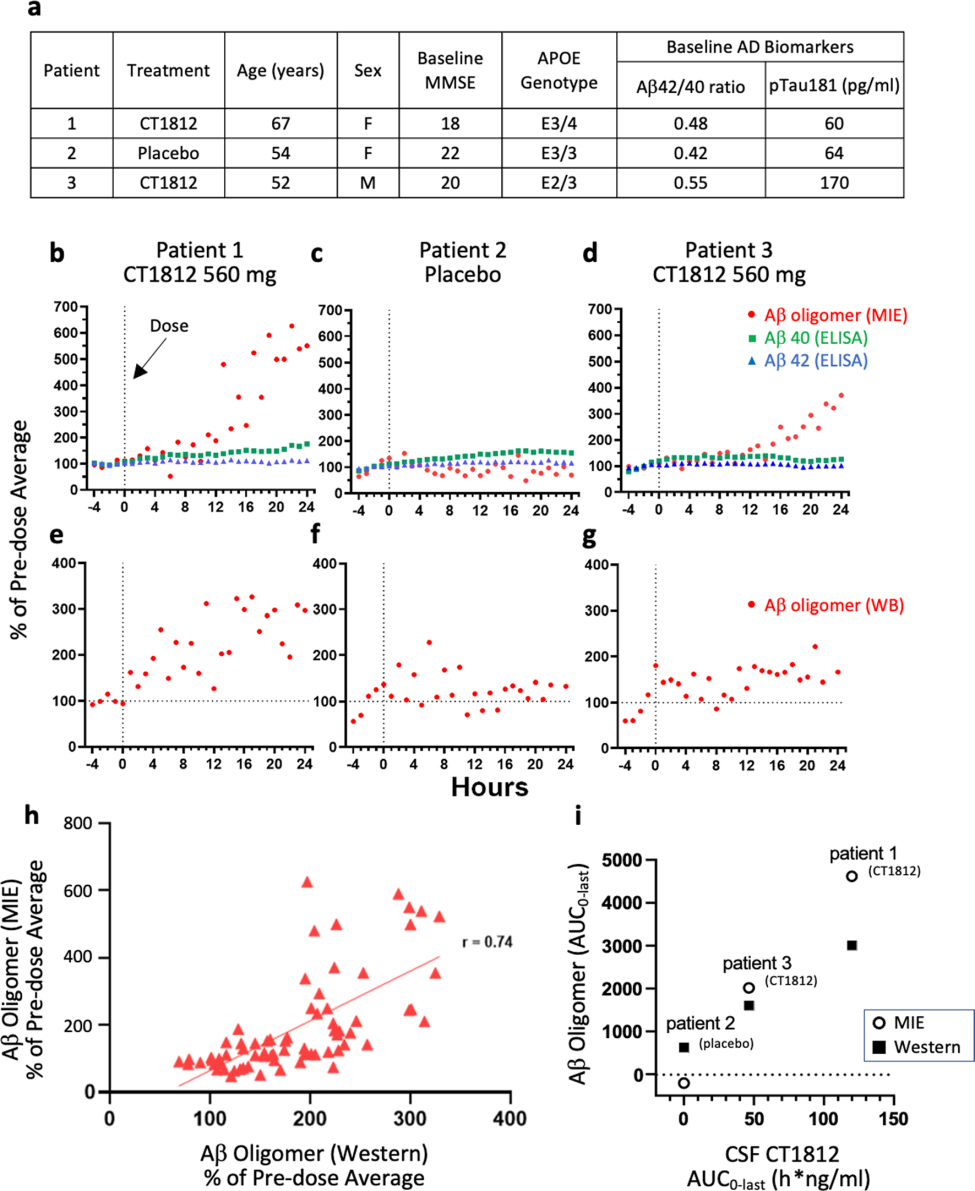
CT1812 treatment results in increases in CSF Aβ oligomers, but not monomers, in an exposure-dependent manner. **a** Subject demographics. **b**–**g** CSF Aβ oligomer or monomer concentrations were measured in individual AD patient samples collected hourly before (− 4 to 0 h as baseline) and 24 h after a single dose of 560 mg CT1812 or placebo. Aβ monomers (ELISA) increased by < 50% of baseline during the post-dose period, but Aβ oligomers (MIE) increased by > 250% (Patient 3) and > 500% (Patient 1) of baseline (**b**–**d**). Non-denaturing western blotting revealed similar increases of CSF Aβ oligomers in CT1812 patients (**e**–**g**). **h** The oligomer level by western blot correlated with that measured by MIE (Pearson* r* = 0.74; *n* = 70 samples) **i** CSF total exposure levels of CT1812 (data shown in Table S1) were related to the total AUC_0-last_ of CSF Aβ oligomer concentration (MIE or Western blot) across patients

To assess target engagement, Aβ oligomer levels in CSF samples were measured at all time points (Fig. 1b–g) using MIE methods similar to those described previously [3], as well as using non-denaturing western blotting quantifying Aβ oligomers ranging from 25 to 99 kDa [3]. CSF Aβ-40 and Aβ-42 monomer levels were also measured at all time points (Lumipulse immunoassay [6]). Plasma and CSF CT1812 concentrations were measured (LC–MS/MS [3]) to understand the pharmacokinetic/pharmacodynamic relationship across the total 24 h of exposure. For each patient, the C_max_ was determined and total drug exposure was calculated as area under the curve (AUC_0-last_).

Given the semi-quantitative nature of the Aβ oligomer assays, the percent change of Aβ oligomer level relative to baseline (average of the pre-dose values) was calculated for each patient (Fig. 1b–g). Aβ oligomer levels in the two CT1812-treated patients, assessed by MIE, increased by > 250%–500% above baseline over time, but did not increase in the placebo-treated patient (Fig. 1b–d). A similar pattern was observed using the independent native gel Western blot method (Fig. 1e–g). A high degree of congruence between these distinct assays was observed (Pearson correlation coefficient *r* = 0.74; Fig. 1h).

The CT1812 exposure level in patient 1 (AUC_0-last_ = 120 h*ng/ml, C_max_ = 24.9 ng/ml) was more than twofold higher than that of patient 3 (AUC_0-last_ = 46.4 h*ng/ml, C_max_ = 7.27 ng/ml, Fig. 1i), allowing for a preliminary gauge of exposure-dependence. Similarly, CSF Aβ oligomer levels were also higher in patient 1 than in patient 3 (Fig. 1i). Together with the preclinical studies [3], this observation is consistent with a drug exposure-dependent impact of CT1812 on Aβ oligomers after a single dose.

Importantly, there was little to no change (< 50% increase from baseline) in Aβ40 and Aβ42 levels over the 24-h period irrespective of treatment. The slight increase observed in some patients may be due to the decreased overall CSF volume caused by multiple CSF collections within a short period [7, 8]. The selective increase in CSF Aβ oligomers but not monomers in the two patients with CT1812 treatment is consistent with preclinical evidence that CT1812 selectively displaces oligomeric over monomeric Aβ [3].

This is the first study evaluating hourly changes of Aβ oligomer concentrations in AD patient CSF and examining how this pharmacodynamic biomarker is impacted by the drug candidate CT1812, which selectively displaces Aβ oligomers allosterically through the S2R. The observed selective increase in CSF Aβ oligomers also suggests that there is a displaceable pool of Aβ oligomers in the AD central nervous system and provides early evidence that the mechanism of action of CT1812 elucidated preclinically translates to the clinic. The observation that the degree of change in Aβ oligomers aligned with the exposure level of CT1812 supports the use of Aβ oligomers as a measure of target engagement in future studies. As the relative increase in Aβ oligomers over time did not reach a plateau by the end of the study, assessment with longer intervals may be warranted to establish the peak time of this pharmacodynamic response. Despite the small cohort size analyzed in this trial, the unique design provides early proof of principle that target engagement can be measured in patients after a single dose of CT1812. Given that toxic Aβ oligomers play a critical role in AD pathogenesis, these findings support continued clinical development of CT1812, which is under further investigation in ongoing phase 2 clinical trials for AD (NCT03507790, NCT04735536) and dementia with Lewy bodies (NCT05225415).

## Supplementary Information

**Additional file 1.** Methods. Table S1: CT1812 pharmacokinetic parameters in plasma and CSF of two AD patients after a single 560-mg oral dose.

## Data Availability

All data needed to evaluate the conclusions are presented here or available upon request.

## Abbreviations

MIE: Microimmunoelectrode
PrP^c^: Cellular prion protein
S2R: Sigma-2 receptor
