# Supplementary material for: A phase 1b randomized clinical trial of CT1812 to measure Aβ oligomer displacement in Alzheimer’s disease using an indwelling CSF catheter
Source: Transl Neurodegener. 2023 May 12;12:24. doi: 10.1186/s40035-023-00358-w (PMC10176668; doi:10.1186/s40035-023-00358-w)
Supplement: Supplementary file 1 — Additional file 1. Methods. Table S1: CT1812 pharmacokinetic parameters in plasma and CSF of two AD patients after a single 560-mg oral dose. [file 40035_2023_358_MOESM1_ESM.docx]

**Additional file 1**

**Methods**

**Study Design**

This study was designed as a multi-center, Phase 1b, randomized, double-blind, placebo controlled parallel-group trial in adults with mild to moderate AD in 18 patients. The study was performed at two separate clinical sites, the University of Pennsylvania (USA) and the Karolinska University Hospital (Sweden). Experienced staff in dedicated in-patient clinical research centers within each institution carried out the trial. The Clinical Pharmacological Trial Unit (CPTU) at the Karolinska Institute is licensed for Phase 1 trials, including first-in-human trials, and certified and regularly inspected by the Swedish Medical Product Agency. The Clinical Translational Research Center (CTRC), at the University of Pennsylvania, is certified for conducting inpatient research. Both centers were subjected to either prequalification or initiation visits to verify the capabilities of the staff and the adequacy of the facilities and equipment.

After consenting to participate in the study, screening procedures were planned to occur between Days -42 and -1. Eligibility criteria for the study included a diagnosis of mild-moderate AD, reflected by a Mini-Mental State Examination (MMSE) score of 18-26, and either an AD-positive amyloid positron emission tomography (PET) scan or an AD biomarker-positive CSF result (low Aβ42 or Aβ42/40 ratio, and high tau or phosphorylated tau (p-tau)) within 12 months before screening). Baseline CSF biomarker cutoffs, using the Fujirebio Lumipulse G assay, were Aβ42/40 ratio <0.72, and pTau-181 >50 pg/ml. Using these criteria, all patients fall within AD biomarker-positive criterion.

Eligible subjects were to be randomized in a 2:1 (active:placebo) ratio and admitted to the study unit on Day -1 for a Confinement Visit to take place over 2 days and consisting of four phases: admission, 28 hour CSF and plasma sampling, a 2-6-hour period of observation, and discharge. Due to difficulties in recruitment, only 3 patients were enrolled before the study was terminated. All three patients were on background therapy and were taking donezapil, but not memantine.

Participants were randomized to receive either a single oral dose of CT1812 (560 mg) or identically appearing placebo capsules. An indwelling catheter was placed in the lumbar subarachnoid space and 4-6 mL CSF samples were collected hourly for 28 hours (five samples pre-dose and 24 samples following drug or placebo administration). Plasma was taken at the same intervals for PK analysis. All measurements and analyses of analyte levels in CSF and plasma were conducted before unblinding samples.

**Safety summary**

There were no deaths. No subjects were withdrawn from the study due to treatment-emergent AEs (TEAEs). One subject (placebo) had SAEs of nausea/vomiting and headache, both severe, and both deemed unlikely related to the lumbar puncture procedure.

**Coating of CSF collection material with human serum albumin (HSA)**

Prior to collection of CSF, all syringes, lumbar needles, catheter lines and collection tubes were coated with sterile, isotonic human serum albumin (HSA). Flexbumin Sterile HSA 25% stock was diluted in Phosphate-Buffered Saline (PBS) to reach the isotonic levels in the CSF (2%). Five baseline and 24 post dose CSF samples were collected in polypropylene tubes coated with isotonic HSA in 500 µL and frozen at -80ºC until analysis. Upon receipt from clinical sites, the samples were thawed and quickly aliquoted into HSA-coated polypropylene tubes and refrozen at -80ºC until analysis.

**Analysis of CSF Aβ oligomers by microimmunoelectrode (MIE) electrochemical assay**

Because Aβ oligomer concentration in the CSF is in the picomolar range and often below limits of quantification for even the most sensitive ELISAs (Savage et al., 2014; Yang et al., 2019), we set out to maximize Aβ oligomer recovery paired with the use of a sensitive oligomer detection assay (Izzo et al., 2021).

Microimmunoelectrodes (MIEs) used to detect Aβ oligomer levels in CSF samples were prepared as previously described (Izzo et al., 2021). Briefly, the oligomer specific antibody A11 (Invitrogen, Cat# AHB0052) was attached to a carbon fiber microelectrode and used to detect concentrations of Aβ oligomers in CSF samples. Following antibody attachment, electrodes were cycled through a warm-up period to check for abnormally shaped voltammetry scans and integrity of the electrode seal before sample testing. Baseline square wave voltammetry scans (SWV) were collected for each electrode before and between samples and were used for background subtraction. Samples from individual patients were randomized and counterbalanced across two sessions within one week of each other. Four 4 replicate scans with 60-second intervals between each sample replicates were performed, with BSA rinses of the electrode between samples. Only MIEs calibrated with increasing concentration of Aβ oligomers that achieved an r2 > 0.80 were included in the analysis. The height of the tyrosine oxidation peak was plotted for each sample after background subtraction Baseline (predose) was determined as the average oxidation peak height for all 5 timepoints taken prior to dosing (-4 to 0 hrs). Oxidation peak heights for each time point were normalized to % predose baseline.

**Measurement of CSF Aβ oligomers by native (non-denaturing) Western blots:**

CSF was analyzed using non-denaturing gel electrophoresis conditions to avoid Aβ assembly and disassembly artifacts, as has been previously described (Izzo et al., 2021). CSF samples were run on 4-15% Tris-HCl nondenaturing gels and Aβ detected with 82E1 monoclonal antibody as previously described (Izzo et al., 2021). Band intensities on the gels were quantified using an Alpha Innotech image system as previously described (Izzo et al., 2021). Samples were analyzed on two separate gels with even numbered collection time points on one gel and odd numbered on another gel. For normalization across gels, all five predose samples were included on both gels. Quantification of the Aβ oligomer intensity values of diffuse bands within 25 kDa to 99 kDa was performed as this range was not confounded by artifactual interference from IgG and hemolyzed blood. For each gel, baseline was determined as the average of the intensities of the five pre-dose samples (-4 to 0 hr). Sample intensity was then normalized as the percent of predose baseline within gel and plotted per patient (Figure 1).

**Pearson correlation analysis of MIE and WB**

In figure 1H, change from baseline values were plotted for all timepoints for which both MIE and WB data were obtained. Because at 23 hours there was no WB data obtained for patients 2 and 3, 70 samples are included: 24 for patient 1, 23 for patient 2, and 23 for patient 3.

**Aβ ELISA measurements**

CSF Aβ40 and Aβ42 monomer levels were measured via the quantitative Lumipulse G β-amyloid 1-40 and 1-42 assay based on CLEIA technology by a two-step immunoassay (Lumipulse G β-amyloid 1-40 or 1-42 Immunoreaction Cartridge; Fujirebio Inc.) on the Lumipulse G System. ELISA values were converted to the percent of the average predose value and the predose average was determined as the average of 5 pre-dose timepoints (-4 to 0 hr). The analyte concentrations for post-dose time points were normalized as the percent of this predose baseline (Figure 1).

**Pharmacokinetic Analysis**

Concentrations of CT1812 in plasma and CSF were quantitated in all samples from the two patients who received active drug using a validated HPLC-MS/MS method with a lower limit of quantitation (LLOQ) of the assay of 0.05 ng/mL in plasma and CSF.

PK parameters were derived by non-compartmental (model independent) methods using Phoenix WinNonlin (Pharmacokinetics, [Gibaldi and Perrier, 1982](#_REFX_58DFB385CA9441FA8E68D5302D6BDE20)) and T_max_, AUC, t1/2, and C_max_ were determined (Supplemental Table 1).

CT1812 concentrations were below the limit of quantification in predose plasma and CSF samples and measurable CT1812 concentrations were observed in all postdose samples assessed for CT1812-treated patients. Following oral administration of a single 560 mg dose, CT1812 was rapidly absorbed with maximum plasma concentrations observed at 1 and 2 hours postdose (T_max_) in Patients 1 and 3, respectively. The appearance of CT1812 in CSF was also rapid, with a 1-hour lag in attainment of maximum concentrations relative to plasma; CSF T_max_ was 2 and 3 hours in Patients 1 and 3, respectively. Thereafter, T_max_, concentrations declined in a biexponential manner in plasma and CSF, with profiles that were parallel in the two matrices.

Concentrations in Patient 1 were consistently higher in plasma and CSF than those in Patient 3 across the time course. C_max_ and AUC_0-last_ values for Patient 1 were 2.8 and 2.6 times higher in plasma and 3.4 and 2.6 times higher in CSF, respectively, than for Patient 3. The extent of penetration into the central nervous system (CNS) was similar in both patients, with CSF/plasma ratios based on C_max_ and AUC_0-last_ values ranging from approximately 0.03 to 0.04.

There was an apparent delay between the time of C_max_ of CT1812 and the maximal pharmacodynamic response (clearance of Aβ oligomers into the CSF); understanding the mechanism of this time lag will be a topic of interest in future studies.

**Statistical analysis**

Because only 3 subjects were enrolled in the study, the group analyses described in the study statistical analysis plan were not feasible. Instead, a post-hoc exploratory analysis was performed after database lock but prior to unblinding. The changes in total Aβ species and for oligomer measurement over time were analyzed on a patient-by-patient basis. For each patient, the percent of predose (-4 to 0 hr) average for each time point and each analyte from 1 hr to 24 hrs post-dose were plotted.

Estimates of the area under the curve for Aβ oligomer concentrations in CSF were calculated for each patient using the percent change from predose baseline (100%) using all 24 time points after dose administration. A negative value indicates net values lower than the predose average.

**Table S1.** CT1812 pharmacokinetic parameters in plasma and CSF of two AD patients after a single 560-mg oral dose

|  |  | **CT1812 pharmacokinetics** | | | | | **Change in Aβ** **Oligomers vs pre-dose** | |
| --- | --- | --- | --- | --- | --- | --- | --- | --- |
| **Matrix** | **Patient** | **C_max_**  **(ng/mL)** | **T_max_**  **(hr)** | **AUC_0-last_**  **(hr*ng/mL)** | **CSF/Plasma Ratio** | | **MIE (AUC)** | **WB**  **(AUC)** |
|  |  |  |  |  | **C_max_^a^** | **AUC_0-last_^b^** |  |  |
| Plasma | 1 | 695 | 1.00 | 3160 | 0.0358 | 0.0381 | - | - |
|  | 3 | 250 | 2.00 | 1210 | 0.0291 | 0.0384 | - | - |
| CSF | 1 | 24.9 | 2.00 | 120 | - | - | 4616 | 3009 |
|  | 3 | 7.27 | 3.00 | 46.4 | - | - | 2012 | 1605 |
|  | 2 |  |  | 0^c^ | - | - | -209 | 627 |
| ^a^based on C_max_; ^b^based on AUC_0-last_; ^c^not measured, inputted as zero for graphing in Figure 1.  AUC_0-last_: area under the curve from time 0 to the last measurable concentration; C_max_: maximum observed concentration; CSF: cerebrospinal fluid; T_max_: time of maximum concentration | | | | | | | | |

**Supplemental References**

Gibaldi M, Perrier D., Eds. Pharmacokinetics (2nd ed.) 1982. CRC Press. https://doi.org/10.1201/b14095

Izzo NJ, Yuede CM, LaBarbera KM, et al. Preclinical and clinical biomarker studies of CT1812: A novel approach to Alzheimer’s disease modification. Alzheimer’s Dement. February 2021:alz.12302. doi:10.1002/alz.12302

Savage MJ, Kalinina J, Wolfe A, et al. A sensitive Aβ oligomer assay discriminates Alzheimer’s and aged control cerebrospinal fluid. J Neurosci. 2014;34(8):2884-2897

Yang T, Dang Y, Ostaszewski B, et al. Target Engagement in an Alzheimer Trial: Crenezumab Lowers Aβ Oligomers in CSF. Ann Neurol. 2019:ana.25513.
